# Apathy progression is associated with brain atrophy and white matter damage in Parkinson’s disease

**DOI:** 10.1093/braincomms/fcaf355

**Published:** 2025-09-13

**Authors:** Roqaie Moqadam, Houman Azizi, Aliza Brzezinski-Rittner, Lucas Alexis Ronat, Shima Raeesi, Alexandru Hanganu, Yashar Zeighami, Mahsa Dadar

**Affiliations:** Department of Medicine, University of Montreal, Montreal, Quebec, Canada H3T 1J4; Department of Psychiatry, Douglas Mental Health University Institute, Montreal, Quebec, Canada H4H 1R3; Centre de Recherche, Institut Universitaire de Gériatrie de Montréal, Montreal, Québec, Canada H3W 1W5; Department of Psychiatry, Douglas Mental Health University Institute, Montreal, Quebec, Canada H4H 1R3; Montreal Neurological Institute and Hospital, McGill University, Montreal, Quebec, Canada H3A 2B4; Integrated Program in Neuroscience, McGill University, Montreal, Quebec, Canada H3A 0G4; Department of Psychiatry, Douglas Mental Health University Institute, Montreal, Quebec, Canada H4H 1R3; Integrated Program in Neuroscience, McGill University, Montreal, Quebec, Canada H3A 0G4; Department of Psychiatry, McGill University, Montreal, Quebec, Canada H3A 1A1; Department of Medicine, University of Montreal, Montreal, Quebec, Canada H3T 1J4; Centre de Recherche, Institut Universitaire de Gériatrie de Montréal, Montreal, Québec, Canada H3W 1W5; Department of Psychiatry, Douglas Mental Health University Institute, Montreal, Quebec, Canada H4H 1R3; Integrated Program in Neuroscience, McGill University, Montreal, Quebec, Canada H3A 0G4; Department of Psychiatry, McGill University, Montreal, Quebec, Canada H3A 1A1; Centre de Recherche, Institut Universitaire de Gériatrie de Montréal, Montreal, Québec, Canada H3W 1W5; Department of Psychology, University of Montreal, Montreal, Quebec, Canada H3T 1J4; Department of Psychiatry, Douglas Mental Health University Institute, Montreal, Quebec, Canada H4H 1R3; Department of Psychiatry, McGill University, Montreal, Quebec, Canada H3A 1A1; Department of Psychiatry, Douglas Mental Health University Institute, Montreal, Quebec, Canada H4H 1R3; Department of Psychiatry, McGill University, Montreal, Quebec, Canada H3A 1A1

**Keywords:** Parkinson's disease, apathy, magnetic resonance imaging, deformation-based morphometry, white matter hyperintensities

## Abstract

Apathy is a prevalent non-motor symptom that significantly impacts the quality of life in Parkinson’s disease (PD) patients. Although previous studies have investigated the neural correlates of apathy in PD, the longitudinal relationships between regional brain atrophy, white matter hyperintensities (WMHs), and apathy progression remain underexplored. Using longitudinal, multisite data of *de novo* PD patients from the Parkinson's Progression Markers Initiative (PPMI), the present study aims to investigate these relationships. We used T1-weighted magnetic resonance imaging (MRI) and clinical data from 445 participants. Apathy was assessed as part of the Movement Disorder Society Unified Parkinson's Disease Rating Scale (MDS-UPDRS) Part I. We applied deformation-based morphometry (DBM) to quantify grey matter atrophy and used the Brain tISsue segmentatiON (BISON) algorithm to segment WMHs from T1-weighted images. Using linear regression models, we performed cross-sectional analyses to identify the associations between baseline brain measurements (DBM and WMH) and apathy severity. Longitudinal analyses utilized linear mixed-effects models to investigate whether baseline brain measurements were associated with future apathy progression over time, accounting for covariates such as age, sex, motion artefacts, Hoehn and Yahr stage, levodopa-equivalent daily dose (LEDD), Total Intracranial Volume (TIV) and baseline apathy. Hypothesis-based and exploratory analyses were conducted to confirm the results previously reported in the literature and explore potential new associations. No cross-sectional regional associations survived multiple comparison corrections. Longitudinal hypothesis-based models confirmed that baseline atrophy in regions such as the bilateral nucleus accumbens area, superior parietal, putamen, insula, left precuneus, right precentral and cerebellum grey matter was significantly associated with future apathy progression. Exploratory longitudinal analyses identified additional regions, including the bilateral lingual, parahippocampal, basal forebrain, ventral diencephalon, isthmus cingulate, thalamus, hippocampus, left middle temporal, right inferior temporal, pericalcarine, medial orbitofrontal, cuneus, where baseline atrophy was correlated with progression of apathy severity. Moreover, greater WMH burden, particularly in the frontal lobe, was associated with worsening apathy. These results highlight the influence of both grey matter atrophy and WMHs on apathy progression in PD.

## Teaser

Moqadam *et al*. report that in de novo Parkinson’s disease patients, baseline grey matter atrophy in regions including the nucleus accumbens, parietal cortex and cerebellum, as well as higher frontal white matter hyperintensities, are associated with future apathy progression. These findings highlight structural brain predictors of worsening apathy over time.

## Graphical Abstract

**Graphical Abstract:**
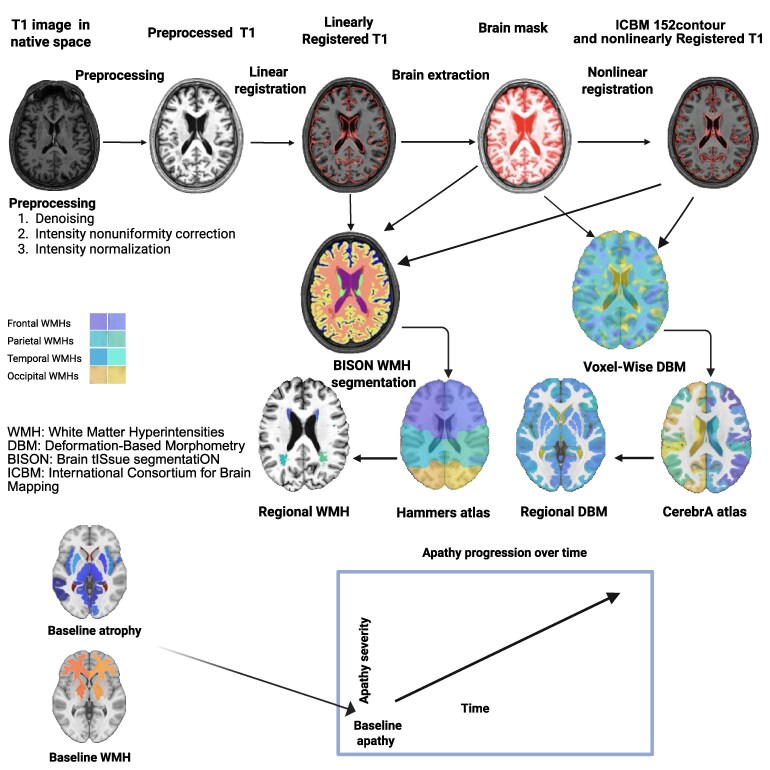

## Introduction

Parkinson's disease (PD) is the second most common neurodegenerative disorder after Alzheimer's disease. In addition to its well-known motor symptoms including tremors, bradykinesia, rigidity and postural instability, PD is associated with a range of non-motor symptoms, such as apathy, depression, anxiety, cognitive impairment, sleep disturbances and autonomic dysfunction.^1,2^ Characterized by a lack of motivation, interest and emotional engagement, apathy is one of the common neuropsychiatric symptoms in PD, substantially impacting the quality of life and independence of the patients.^2-5^

Stuss and colleagues define three distinct subtypes of apathy, including behavioural, motivational and cognitive apathy, each associated with specific neural circuits.^6-8^ While inferential, identifying these subtypes in PD populations is important, as it can lead to a more precise understanding of how different patterns of brain atrophy may be associated with specific dimensions of apathy. For example, motivational apathy, which is a loss of drive and initiative, is associated with dysfunctions in the ventral striatum and its reward-processing circuits, leading to dopaminergic and serotonergic dysfunction in the mesocorticolimbic pathway.^6,7^ Behavioural apathy or reduced self-initiated actions is associated with atrophy in the medial prefrontal cortex, anterior cingulate cortex and supplementary motor area, resulting in impaired action initiation and goal-directed behaviour. Finally, cognitive apathy or difficulty in planning and decision-making is related to dysfunctions in the dorsolateral prefrontal cortex and frontostriatal circuits, causing impairments in executive functions, working memory and cognitive flexibility.^6,7^ However, these associations are not completely distinct and mutually exclusive. For example, behavioural apathy as reflected in reduced self-initiated action has overlaps with motivational circuitry, and lower dopamine levels have been implicated in action initiation.^6,7^

While apathy is also present in up to 30% of healthy older adults, the apathy observed in PD patients is believed to differ from that associated with healthy ageing.^6,9^ This difference is primarily due to its association with specific neurotransmitter dysfunctions and alterations in apathetic PD patients.^10^ As such, PD apathy is associated with dopaminergic deficits and disruptions in frontostriatal circuits. These dysfunctions negatively impact reward processing, intrinsic motivation and emotion regulation.^6,10,11^ Structural changes in areas such as the basal ganglia and frontal cortex are thought to contribute to these deficits, emphasizing how PD-related apathy might differ from the aging-related alterations in motivation and engagement.^6^

The literature reports varying results in the prevalence of apathy across different PD populations, with a wide range from 13.9% to 70% depending on different factors, including demographics, diagnostic criteria and assessment tools, disease stage and treatment.^3,4,12^ While apathy can emerge at any stage of the disease, it is frequently present in earlier disease stages.^6^ In a recent review of the non-motor aspects of PD symptomatology, apathy was recognized as a distinct feature, occurring several years before the motor symptoms became evident.^2^ In a cohort of 109 newly diagnosed, untreated PD patients, apathy was commonly reported during the two-year period preceding the motor symptoms, with approximately 11% of the cohort identifying its onset within the period preceding the motor symptoms.^13^

Previous neuroimaging studies in cross-sectional samples have reported associations between cortical and subcortical atrophy and apathy in PD patients.^14-23^ These studies vary in their approach to evaluating apathy, using either presence or severity measures. Regarding the presence of apathy, several studies used the Neuropsychiatric Inventory (NPI)^24^ and Lille Apathy Rating Scale (LARS)^25^ to identify brain regions associated with apathy. These studies consistently reported associations between apathy presence and atrophy in regions such as the anterior cingulate cortex, nucleus accumbens and caudate, emphasizing the involvement of frontostriatal circuits in the presence of apathy.^15-21^ Studies focusing on apathy severity often utilize the Starkstein Apathy Scale (SAS),^26^ Apathy Evaluation Scale (AES-I)^14,27^ and NPI. These studies established correlations between the severity of apathy and atrophy in certain brain regions, including the inferior parietal lobule, orbitofrontal cortex and insula, underscoring the relationship between cortical degeneration and apathy severity^14,15,19,20,22,23^ (Table 1). Longitudinal assessments of apathy related to brain atrophy have been more scarce. Morris *et al*.^21^ reported an association between the nucleus accumbens volume and the presence of apathy over 2 years of follow-up. In contrast, Ranjbar *et al*.^23^ reported a correlation between lower atrophy in the right putamen and increased apathy severity over time.

**Table 1:** Regions linked to apathy in PD based on previous literature

Region name	References	Sample size (disease stage)	Apathy assessment	Presence/Severity	Covariates
Middle Frontal	Alzahrani *et al*.^16^	65 (Mild)	NPI	Presence	Age, Education, Sex, TIV
	Rashidi-Ranjba *et al*.^23^	137 (Early to moderate, MoCA > 19)	NPI	Severity	Age, Sex, Education
Nucleus Accumbens	Morris *et al*.^21^	199 (HY∼ 1–3)	NPI	Presence	Age, Sex, UPDRS-III, MoCA
	Carriere *et al*.^15^	20 (non-demented, non-depressed)	LARS	Severity	LEDD, DD
	Martinez-Horta *et al*.^17^	36 (early to mid, HY: 1.8 ± 0.4)	UPDRS-I*	Presence	Age, Sex, TIV
Lateral Orbitofrontal	Martinez-Horta *et al*.^17^	36 (early to mid, HY: 1.8 ± 0.4)	UPDRS-I*	Presence	
Rostral Anterior Cingulate	Alzahrani *et al*.^16^	65 (mild)	NPI	Presence	
	Morris *et al*.^21^	199 (HY: 1–3)	NPI	Presence	
	Prange *et al*.^20^	27 (De novo)	LARS	Presence	Age, Sex
Superior Parietal	Martinez-Horta *et al*.^17^	36 (early to mid, HY: 1.8 ± 0.4)	UPDRS-I*	Presence	
Inferior Parietal	Reijnders *et al*.^14^	55 (H&Y: 1.5–3)	LARS	Severity	Age, MMSE, GM volume
	Martinez-Horta *et al*.^17^	36 (early to mid, HY: 1.8 ± 0.4)	UPDRS-I*	Presence	
Putamen	Ye *et al*.^19^	207 de novo ∼ MCI	NPI	Presence	Age, Sex, education, TIV
Insula	Alzahrani *et al*.^16^	65 (Mild)	NPI	Presence	
	Reijnders *et al*.^14^	55 (HY: 1.5–3)	LARS	Severity	
	Ye *et al*.^19^	207 (de novo ∼ MCI)	NPI	Presence, Severity	
	Rashidi-Ranjba *et al*.^23^	137(early to moderate, MoCA > 19)	NPI	Severity	
Precuneus	Reijnders *et al*.^14^	55 (HY: 1.5–3)	LARS	Severity	
Precentral	Alzahrani *et al*.^16^	65 (Mild)	NPI	Presence	
	Reijnders *et al*.^14^	55 (H&Y: 1.5–3)	LARS	Severity	
Caudal Midbrain	Prange *et al*.^20^	27 (de novo)	SAS	Severity	
Inferior Frontal	Alzahrani *et al*.^16^	65 (mild)	NPI	Presence	
	Martinez-Horta *et al*.^17^	36 (early to moderate, HY: 1.8 ± 0.4)	UPDRS-I*		
	Reijnders *et al*.^14^	55 (HY: 1.5–3)	LARS	Severity	
Superior Temporal	Alzahran *et al*.^16^	65 (mild)	NPI	Presence	
	Prange *et al*.^20^	27 (de novo)	LARS	Presence	
	Rashidi-Ranjba *et al*.^23^	137 (early to moderate, MoCA > 19)	NPI	Severity	
Cerebellum GM	Prange *et al*.^20^	27 (de novo)	SAS	Severity	
Caudate	Morris *et al*.^21^	199 (HY∼ 1–3)	NPI	Presence	
	Carriere *et al*.^15^	20 (non-demented, non-depressed)	LARS		
No regions	Lucas-Jiménez *et al*.^18^	32 (HY ≤ 3)	LARS	Presence	TIV, UPDRS-III, MCI
No regions	Sampedro *et al*.^22^	44 (DD: 9 yrs, relatively high UPDRS-III)	SAS	Severity	Age, sex, education, DD LEDD, UPDRS-III

LARS, Lille Apathy Rating Scale; SAS, Starkstein Apathy Scale; NPI, Neuropsychiatric Inventory; UPDRS, Unified Parkinson's Disease Rating Scale; DD, disease duration; HY, Hoehn and Yahr; TIV, total intracranial volume; MCI, mild cognitive impairment; LEDD, levodopa-equivalent daily dose; GM, grey matter; MoCA, Montreal Cognitive Assessment.

^*^Item 4, Apathy Score 2/3.

Beyond associations with grey matter atrophy, apathy has been linked to spatially extensive reductions in white matter (WM) integrity.^28^ Using diffusion-weighted imaging (DWI), Wen *et al*.^29^ found a decrease in WM network efficiency and connectivity related to apathy in PD, particularly within the frontal, temporal, parietal and basal ganglia regions. However, DWI is not frequently used clinically, particularly in the context of PD.^30^ On the other hand, in normal aging and individuals with Alzheimer’s disease,^28,31^ the literature reports significant associations between apathy symptoms and increased volume of white matter hyperintensities (WMHs) in frontal and temporal lobes, areas critical for motivation and emotional regulation.^28^ WMHs are areas of increased signal on fluid-attenuated inversion recovery (FLAIR) sequences and are markers of cerebrovascular pathology.^32^ WMHs are also commonly present in patients with PD.^33^ The association between WMHs and apathy in PD remains understudied, with a single study in a cohort of 141 PD patients reporting a significant link between WMH and apathy, where WMH severity was a strong predictor of apathy severity and progression in the patients.^30^ However, this study used the visual Fazekas scale for WMH assessment,^34^ which does not reflect the full extent of WMH burden. In addition, only the global WMH rating was utilized, and the study did not assess how WMHs in different brain regions contribute to apathy.^30^

Although neural correlates of apathy have previously been examined, longitudinal relationships between regional cerebral atrophy, WMHs and apathy require further investigation. The present study takes advantage of the large sample size and the availability of longitudinal clinical data from the Parkinson's Progression Markers Initiative (PPMI) dataset to examine the relationship between apathy, regional brain atrophy and WMH in patients with PD. We hypothesize that regional brain atrophy and WMH are associated with the severity and future progression of apathy in PD patients.

## Materials and methods

### Data

T1-weighted MRI and clinical data from 546 participants with PD were obtained from the Parkinson's Progression Markers Initiative (PPMI) database (www.ppmi-info.org/data).^35^ The PPMI is a longitudinal, multisite observational study of newly diagnosed and untreated patients with PD across the United States and Europe. Clinical assessments included Hoehn and Yahr stage (HY Stage), and apathy scores based on the Movement Disorders Society Unified Parkinson’s Disease Rating Scale part I (MDS-UPDRS part I).^35,36^ Levodopa-equivalent daily dosage (LEDD) was calculated based on the dosage and type of the dopaminergic medications taken at the time of each visit according to the guidelines provided by the PPMI (https://www.ppmi-info.org/ Data User Guide.pdf). Clinical and demographic data were organized and processed through our open-source data wrangling pipeline (https://github.com/HoumanAzizi/PPMI_Data_Wrangling). Participants who had received medication at baseline or had missing MRI, demographics, or clinical information were excluded from the present study.

### MRI pre-processing

**Figure 1:**
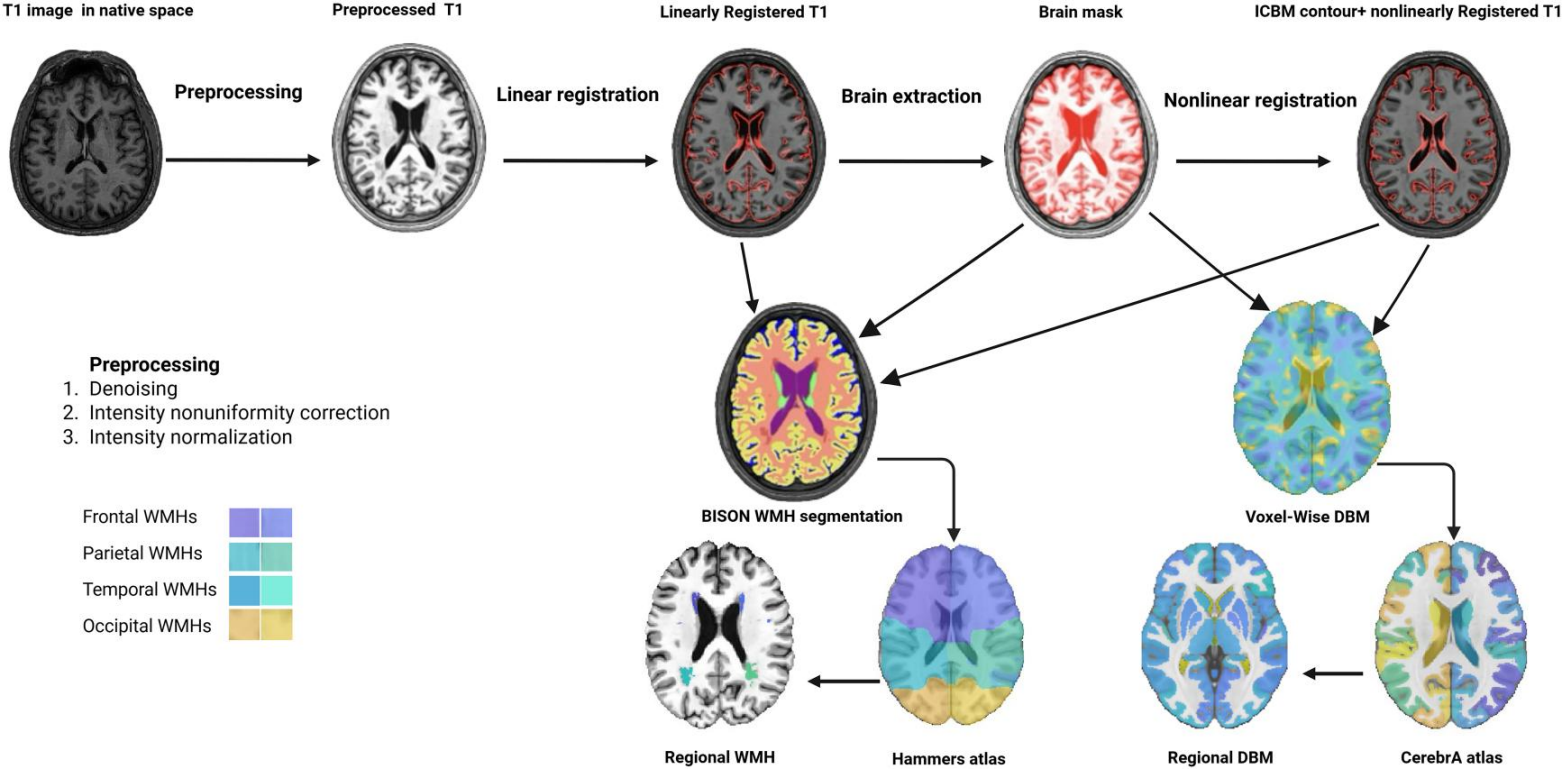
**Flow diagram of the MRI processing steps performed.** Native T1-weighted images were preprocessed (denoising, non-uniformity correction and intensity normalization) and linearly registered to the MNI-ICBM152. Nonlinear registration was performed following brain extraction to derive voxel-wise DBM maps. CerebrA atlas was then used to extract regional DBM measures. The outputs of the linear registration, brain mask and nonlinear registration steps were also used by BISON to derive voxel-wise WMH segmentations. Hammers’ lobar atlas was used to extract regional WMH measures. WMH, white matter hyperintensities; DBM, deformation-based morphometry; MNI, Montreal Neurological Institute; ICBM, International Consortium for Brain Mapping.

Figure 1 provides a schematic of the MRI processing steps performed. T1-weighted MRIs were processed using our open-source image processing pipeline based on the open-access MINC-Toolkit v2 (https://bic-mni.github.io/) and Advanced Normalization Tools (ANTs) (https://stnava.github.io/ANTs/) toolkits (https://github.com/VANDAlab/Preprocessing_Pipeline). Pre-processing steps included noise reduction,^37^ intensity non-uniformity correction,^38^ and intensity normalization into the range [0–100]. The preprocessed T1-weighted images were first linearly^39^ and then nonlinearly^40^ registered to the MNI-ICBM152 average template.^41^ All the image processing steps (i.e. pre-processing, linear and nonlinear registrations) were visually quality controlled by an experienced rater (R.M.) blind to the clinical diagnosis, and the cases that did not pass this quality control step (*N* = 44) were excluded from the subsequent analyses.

### Deformation-based morphometry (DBM)

Deformation-based morphometry **(**DBM) was used to extract regional grey matter atrophy, calculated as the Jacobian determinant of the deformation field from the nonlinear transformations.^42^ DBM values reflect the relative volume of each voxel compared to the MNI-ICBM152 template, i.e. a value of 1 denotes the same volume compared to its equivalent voxel in the template, a value below 1 denotes a smaller volume than the corresponding voxel in the template, and a value above 1 denotes a larger volume than the corresponding voxel in the template. Therefore, atrophy for a particular region can be inferred from a decrease in DBM values. Average grey matter atrophy in 102 cortical and subcortical regions was determined using the CerebrA atlas.^41^

### WMH measurements

BISON,^43^ a previously validated automatic segmentation tool, was used to segment the WMHs. BISON combines a Random Forest classifier with a collection of location and intensity features obtained from a library of manually segmented scans to detect WMHs (Fig. 1). Since the PPMI dataset lacked consistently acquired T2-weighted and fluid-attenuated inversion recovery (FLAIR) images for all participants, T1-weighted images were utilized for WMH segmentation. Although FLAIR and T2-weighted images are ideal for performing WMH assessments, in our previous work, we have shown that T1w-based segmentations from BISON yield estimates with strong correlations with FLAIR-based segmentations (*r* = 0.96),^44^ and have been previously used to assess WMH burden based on T1w images in multi-center cohorts, including the PPMI.^45-47^ The WMH segmentations were visually quality controlled by an experienced rater (R.M.), and cases that did not pass this quality control step (*N* = 7) were excluded from the analyses. The number of voxels designated as WMH (measured in mm^3^) in the standard space (i.e. adjusted for intracranial volume) in each brain lobe and hemisphere (based on Hammer’s lobar atlas^44,48^) as well as across the entire brain were used to define regional and global WMH volumes, respectively.^44,49^ WMH volumes were log-transformed to obtain a normal distribution.

### Motion assessment

**Figure 2:**
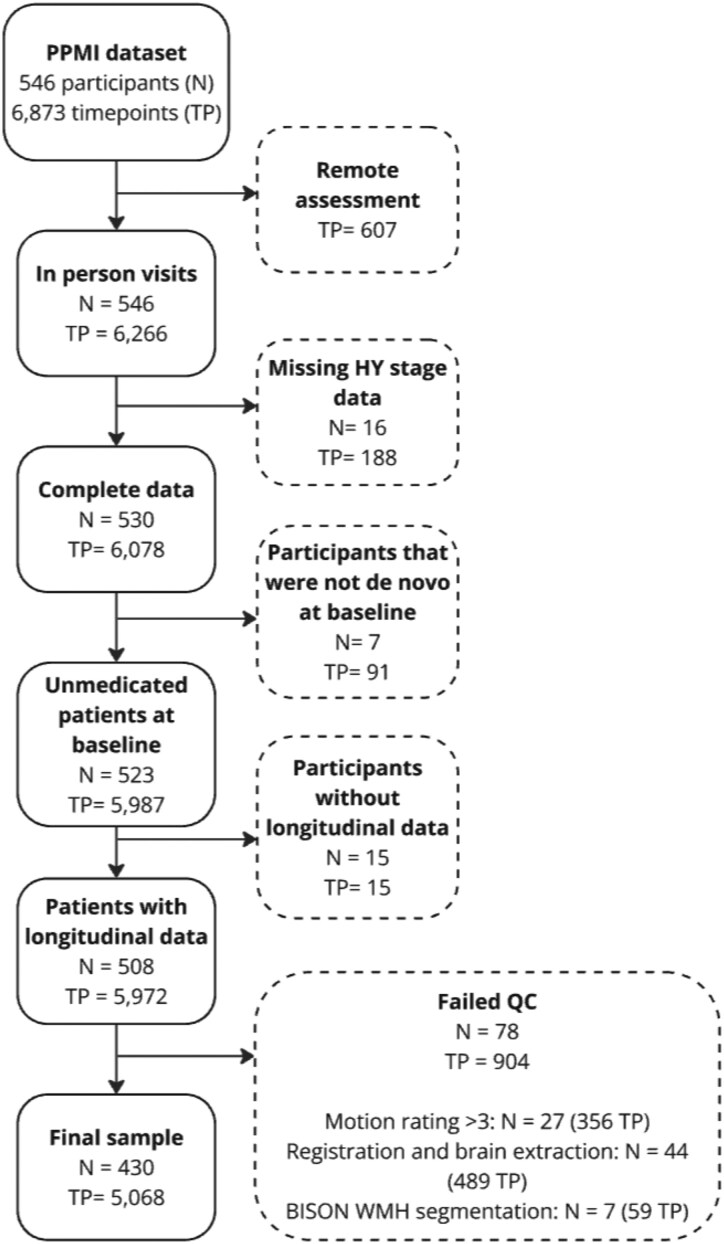
**Diagram summarizing the participant selection procedure.** A total of 116 participants were excluded due to the unavailability of clinical assessments, the presence of MRI artefacts, or failure of image processing steps, leading to a final sample of 430 de novo PD patients with 5068 longitudinal clinical assessments. TP, Time point. QC, Quality control. HY, Hoehn and Yahr.

Previous research, including ours, has shown a subtle but consistent impact of motion on grey matter atrophy estimations.^50-52^ As such, all the images that passed the QC steps outlined in the previous steps further underwent a visual inspection process to assess the presence and severity of motion similar to our previous work (R.M.).^52^ Based on our previous findings, cases with motion severity ratings greater than 3 (N = 27) were excluded.^52^ Motion severity scores were further used as covariates in all models^52^ (Fig. 2).

### Apathy assessment

Apathy was assessed using the Unified Parkinson's Disease Rating Scale (UPDRS) part I with the following criteria: 0 represents no apathy, 1 indicates slight apathy, when the patient experiences apathy less than one day per week, 2 denotes mild apathy, when the patient experiences apathy more than one day per week but less than half of the days, 3 indicates moderate apathy, when the patient feels apathetic more than half of the days, 4 corresponds to severe apathy where the patient feels apathetic all the time.^36^

### Statistical analyses

#### Cross-sectional analyses

The following linear regression models were used to investigate the cross-sectional relationships between brain measurements (i.e. regional DBM and WMH values) and apathy scores at baseline.


Apathy∼BrainMeasure+Age+Sex+Motionrating+TIV+HYStage

The variable of interest was *Brain Measure*, indicating the associations between the brain measures (i.e. regional DBM and WMHs) and apathy after accounting for covariates. Age, sex, Total Intracranial Volume (TIV),^53^ motion rating and HY Stage were included as covariates in the models.

#### Longitudinal analyses

A series of mixed-effect models were used to investigate the relationship between baseline brain measures and longitudinal changes in apathy scores. The following linear mixed-effects models were conducted to examine whether regional DBM, total, or regional WMH would influence the apathy score:


$$\begin{array}{r} \begin{array}{c} Apathy∼Brain\;Measure\;Baseline+Years\;from\;Baseline\\ +Brain\;Measure\;Baseline:Years\;from\\ Baseline+Age\;Baseline+Sex\\ +Motion\;rating\;Baseline+Apathy\;Baseline+TIV\\ +LEDD+HYStage+(1|Patient\;ID) \end{array} \end{array}$$

The variable of interest was the interaction term *Brain Measure:Years from Baseline*, reflecting how baseline atrophy and WMHs contribute to future apathy in PD. Age at baseline, sex, motion rating (corresponding to the baseline MRI), HY Stage, baseline apathy scores, TIV and LEDD were included as covariates. Patient ID was included as a categorical random effect.

### Hypothesis-based and exploratory analyses

The DBM analyses were performed at two levels: (i) hypothesis-based, assessing the impact of atrophy in brain regions reported to be associated with apathy in PD in the literature,^14-23^ and (ii) exploratory, assessing the potential impact of atrophy in all CerebrA GM regions to determine if any new regions could be identified. Table 1 presents the list of the regions included in the hypothesis-based analyses and whether their atrophy was related to apathy presence or severity from the literature. Similar analyses were also completed for global as well as regional WMHs. All analyses (i.e. GM and WMHs) were corrected for multiple comparisons using the False Discovery Rate (FDR) controlling technique.^54^ All statistical analyses were performed using MATLAB version 2022a. fitlm and fitlme functions were used to complete the linear regression and mixed-effects models, respectively.

To ensure the results were not influenced by other neuropsychiatric comorbidities, use of psychotropic medications or vascular risk factors, the analyses were repeated, including anxiety (measured by total State-Trait Anxiety Inventory [STAI] scores), depression (measured by Geriatric Depression Scale [GDS] scores), vascular risk factors (including BMI [Body Mass Index], hypertension, orthostatic hypotension, hypercholesterolaemia, dyslipidaemia, hyperlipidaemia and diabetes) and use of psychotropic medications (Clonazepam, Citalopram, Desvenlafaxine, Seroquel, Lorazepam, Sertraline, Bupropion, Mirtazapine, Amitriptyline, Escitalopram, Zopiclone, Zoloft, Venlafaxine, Quetiapine, Alprazolam, Duloxetine, Aripiprazole, Diazepam, Trazodone, Loxapine, Fluoxetine, Risperidone, Clomipramine) as covariates in the models.

## Results

Figure 2 summarizes the results of the quality control and participant selection process. The baseline and longitudinal demographics and clinical characteristics of the PD patients included in this study are summarized in Table 2. Among all PD participants in the PPMI, our study focused on the subset of 546 individuals with idiopathic PD who had T1w MRI available at the initial visit, excluding genetic variants and SWEDD cases. A total of 445 PD patients had baseline MRI and clinical assessments available, with ages between 33.8 and 84.9. Additionally, 430 participants had longitudinal clinical assessments available, resulting in a final longitudinal dataset of 430 patients with 5068 timepoints. There were no significant differences in demographic and clinical characteristics of the included patients and those that were excluded based on MRI QC (Supplementary Table 1).

**Table 2:** Characteristics of the participants included in this study

Measure	Baseline-only sample	Longitudinal sample at baseline	Longitudinal timepoints
N (Visit)	445 (445)	430 (430)	430 (5068)
Sex (%female)	150(33.7)	145(33.7)	1676 (33.07)
Age	62.42 ± 9.62	62.27 ± 9.63	64.38 ± 9.88
Years from Baseline	-	-	3.21 ± 3.1
Education Years	15.99 ± 2.99	16.01 ± 2.97	15.88 ± 2.85
MOCA	27.10 ± 2.26	27.01 ± 2.48	26.75 ± 3.03
LEDD	-	-	364.15 ± 388.41
Apathy Score	0.24 ± 0.57	0.23 ± 0.57	0.35 ± 0.68
GDS	2.30 ± 2.48	2.27 ± 2.47	2.60 ± 2.76
STAI	63.91 ± 18.12	63.71 ± 18.1	64.69 ± 18.33
BMI	26.78 ± 4.78	26.56 ± 4.81	26.52 ± 4.86
HY Stage	1.61 ± 0.50	1.62 ± 0.50	1.87 ± 0.55
UPDRS-III	21.41 ± 8.96	21.39 ± 9.09	25.94 ± 12.08

MoCA, Montreal Cognitive Assessment; LEDD, levodopa-equivalent daily dose; GDS, Geriatric Depression Scale; STAI, State-Trait Anxiety Inventory; BMI, Body Mass Index; HY, Hoehn and Yahr; UPDRS, Unified Parkinson's Disease Rating Scale.

### Hypothesis-based analyses

**Figure 3:**
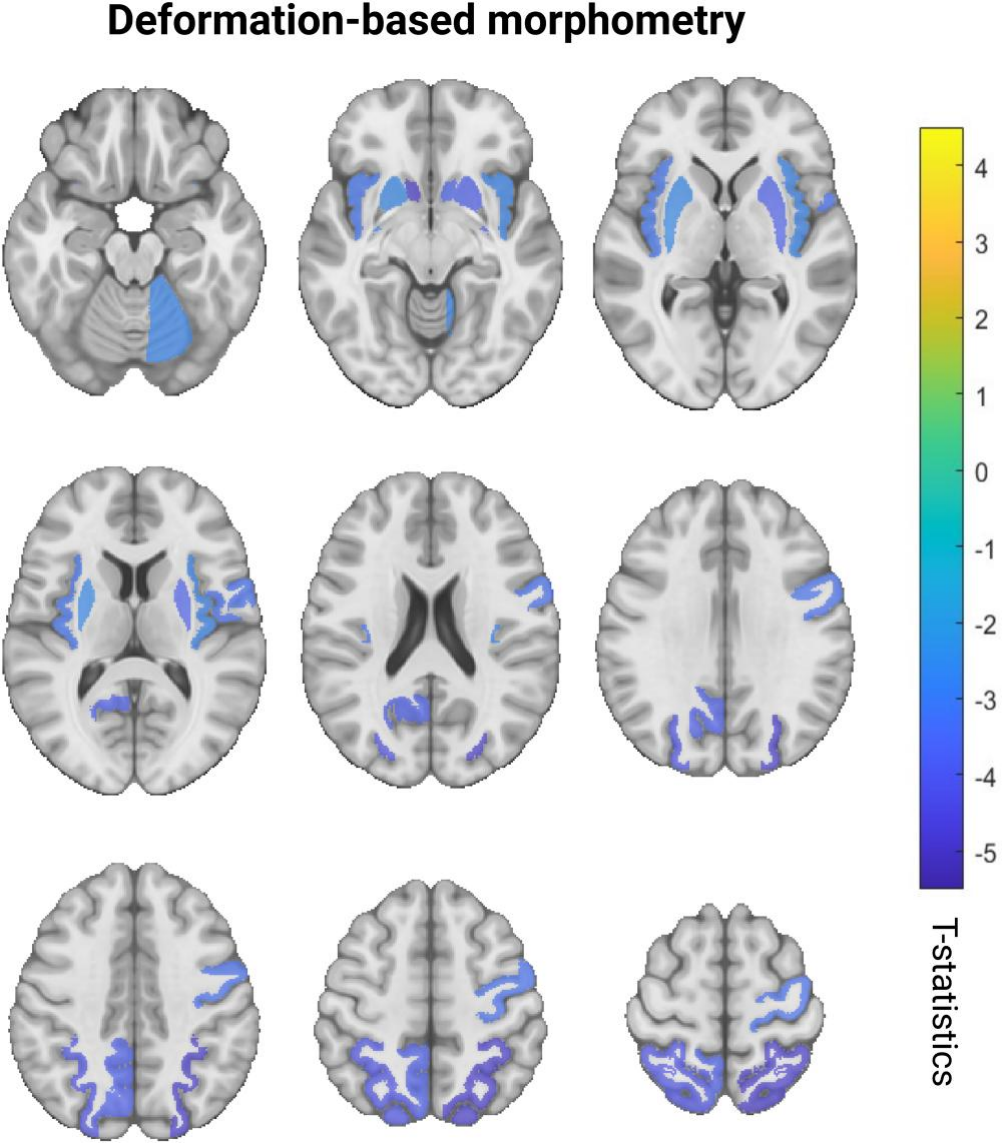
**Longitudinal hypothesis-based analyses results.** Cold colours indicate the *t*-statistics values corresponding to the significant longitudinal associations between regional atrophy and severity of future apathy after FDR correction. Statistical analyses were performed using data from 430 PD participants with 5068 longitudinal timepoints for clinical assessments. Only regions identified a priori based on previous literature were tested in this analysis. FDR, false discovery rate; PD, Parkinson’s disease.

No cross-sectional regional associations survived multiple comparison corrections. The longitudinal hypothesis-based models revealed significant interaction effects between baseline atrophy (i.e. lower DBM values) and time in relation to apathy in the bilateral accumbens area, superior parietal, putamen, insula, left precuneus, right precentral and cerebellum grey matter. (Figure 3, Supplementary Table 2).

### Exploratory analyses

**Figure 4:**
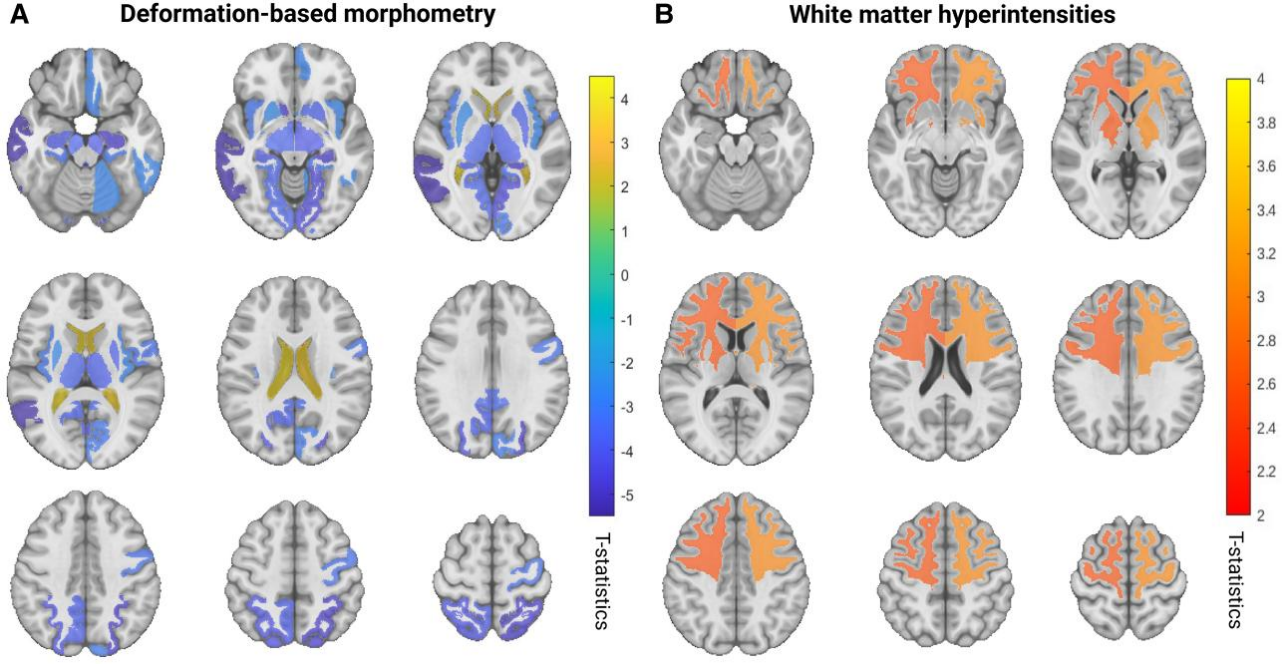
**Longitudinal exploratory analysis results.** (**A**) Cold colours indicate the *t*-statistics values corresponding to the significant longitudinal associations between baseline regional atrophy and severity of future apathy after FDR correction. Warm colours indicate the significant longitudinal associations between baseline ventricular expansion and severity of future apathy after FDR correction. (**B**) Colours indicate the *t*-statistics values corresponding to the significant longitudinal associations between baseline WMH burden and severity of future apathy after FDR correction. All analyses were performed using data from 430 PD participants, with 5068 longitudinal timepoints for clinical assessments. FDR, False Discovery Rate. PD, Parkinson’s disease. WMH, white matter hyperintensities.

No cross-sectional regional associations survived multiple comparison corrections in the exploratory analyses for either DBM or WMH measurements. Longitudinal exploratory analyses further revealed significant interactions between baseline atrophy and time impacting future apathy in the bilateral lingual, parahippocampal, basal forebrain, ventral diencephalon, isthmus cingulate, thalamus, hippocampus, the left middle temporal and right hemisphere, including the inferior temporal, pericalcarine, medial orbitofrontal and cuneus. These results suggest that baseline atrophy in these regions may impact the longitudinal trajectory of apathy in PD (Fig.4A, Supplementary Table 3). Longitudinal assessment of WMHs also showed significant interactions of bilateral frontal lobe WMH burden at baseline and time impacting future apathy (Fig.4B, Supplementary Table 4).

Repeating the analyses with anxiety and depression scores, psychotropic medications and vascular risk factors as covariates yielded similar results in terms of atrophy patterns and significance of the regions (Supplementary Table 5). The analyses were also repeated considering apathy as an ordinal variable with the same covariates, yielding similar results (Supplementary Fig. 1)

## Discussion

The present study investigated the underlying neural correlates of apathy in PD by utilizing a large dataset with longitudinal apathy assessments. Our findings emphasize the importance of specific brain regions in the manifestation of apathy in de novo PD. Our cross-sectional analyses did not reveal any associations between apathy severity and regional atrophy at baseline when patients were at the de novo stage. Utilizing baseline DBM data and multiple apathy measurements per participant, our longitudinal mixed-effects modelling analyses confirmed significant associations between future apathy and baseline atrophy in the bilateral accumbens area,^15,17,21^ superior parietal,^17^ putamen,^19^ insula,^14,16,19,23^ left precuneus,^14^ precentral^14,16^ and right cerebellum GM.^20^

To further examine the relationship between brain structural changes and apathy severity, we conducted exploratory analyses using cross-sectional and longitudinal approaches. While we found no regions to show significant associations with apathy severity cross-sectionally, our longitudinal exploratory analysis revealed significant associations of atrophy in several regions with changes in apathy severity over time. This highlights the importance of considering disease progression in understanding the neural underpinnings of apathy in PD. The increased statistical power provided by our large sample size allowed us to extend previous findings in the literature regarding the association between brain atrophy and apathy in PD. The absence of significant results in cross-sectional analyses suggests that the relationship between atrophy and apathy may be weak at baseline de novo stages and become more apparent with disease progression, and therefore can be identified using a longitudinal framework. Our study revealed additional regions linked to apathy in PD, providing a more comprehensive understanding of the mechanisms underlying apathy in PD.

In the PPMI, apathy was only assessed using MDS-UPDRS-I, and as such, we did not have access to more comprehensive measures of apathy such as the LARS. Indeed, other more comprehensive scales, including the LARS, SAS, or NPI, would have offered richer and more multidimensional assessments of apathy.^11,55^ While this information was not available in the PPMI dataset, previous research in other datasets has validated the use of the MDS-UPDRS apathy item as an effective tool for assessment of apathy.^56,57^ For example, Weintraut *et al*.^57^ and Gallagher *et al*.^56^ demonstrated significant correlations between UPDRS-1.Apathy item and well-established apathy scales, LARS, with Spearman's ρ values ranging between 0.516 and 0.656, *P* < 0.001. Based on these results, notwithstanding the fact that MDS-UPDRS-I.Apathy scores lack the precision and depth of apathy assessment scales such as the LARS, SAS, AS and NPI, they still can provide good estimates of the severity of apathy in PD. Furthermore, employing scales that distinguish apathy related to depression, executive dysfunction/cognitive impairment, emotional blunting/reduced reward sensitivity, or auto-activation deficits/athymhormia could potentially identify specific brain regions associated with each component and improve the identification of neural predictors of apathy presence and severity over time.

Associations between apathy and atrophy in regions such as the accumbens, putamen, insula, thalamus and hippocampus suggest contributions to the motivational apathy subtype in this sample. This subtype is characterized by diminished reward sensitivity and reduced intrinsic motivation.^6,11^ In addition, this interpretation is supported by atrophy in the parahippocampal, ventral diencephalon and cingulate isthmus, implicating these regions in reward processing and emotional regulation.^6,11^ It is worth noting that in areas related to functional impairment such as the medial frontal cortex, orbitofrontal cortex and caudate nucleus, no significant relationship was found between the progression of apathy and atrophy.^6^ This suggests that cognitive apathy, associated with executive dysfunction, is less prominent in this group of PD patients.^6^ These observations support the idea that motivational apathy, which is associated with deficits in reward processing and goal-directed behaviour, may be the dominant subtype in this early-stage PD group of patients. The lack of significant associations in prefrontal regions typically implicated in cognitive apathy, such as the dorsolateral prefrontal cortex, may reflect the relatively preserved executive function in this de novo sample.

Our findings have important implications in terms of patient management and treatment. Distinguishing apathy subtypes is not only conceptually important but also critical for interpreting longitudinal trajectories of neurodegeneration and symptom progression in PD. From a therapeutic perspective, interventions that target dopaminergic systems are more effective for behavioural and motivational apathy.^6,7^ As such, dopaminergic treatments may improve goal-directed behaviour and reward sensitivity and reduce symptoms in these patients.^6,7^ In contrast, treatments that target executive dysfunction, such as cholinergic therapies or cognitive rehabilitation, may be less relevant for this group.^6,7^ This reinforces the importance of recognizing apathy subtypes in PD and tailoring interventions accordingly.

Utilizing quantitative volumetric assessments, we found that frontal lobe WMH burden at baseline is strongly associated with the increase in apathy scores over time. Our lobar WMH segmentation allows us to examine the correlation of WMH burden with apathy progression both at the whole brain level and within specific brain lobes. This approach provided a more precise understanding of brain changes associated with apathy, which was previously unexamined.^30^ Our results suggest that early WMH burden at the frontal lobes is significantly associated with worsening apathy in the future. As WMHs can be managed (e.g. through antihypertensive medications), targeting vascular pathology early might offer a therapeutic avenue to mitigate the progression of apathy in PD patients.^32^ This suggests the importance of incorporating WMH burden assessments in routine clinical evaluations to better predict and manage apathy in PD.

The image processing pipelines used in this study have been specifically developed and extensively validated for use in multi-center and multi-scanner applications, and have previously been employed in numerous studies to investigate atrophy and WMH burden in aging^58^ and different neurodegenerative disorders such as Alzheimer’s disease,^45,59,60^ frontotemporal dementia,^61,62^ amyotrophic lateral sclerosis,^63,64^ multiple sclerosis^65^ and PD.^66-70^ Further still, all image processing steps were quality controlled to ensure the accuracy of the derived atrophy and WMH measurements. Our study also has limitations that need to be taken into account. While the UPDRS-I is used in the PPMI dataset, its accuracy in assessing apathy is limited compared to other, more comprehensive apathy assessment tools. In this regard, Pagonabarraga *et al*.^11^ highlight that while the UPDRS-I is useful for screening purposes, it might lack the depth and specificity needed to assess apathy at PD. In contrast, scales such as the SAS and the LARS are more robust in assessing apathy as they investigate a more comprehensive set of dimensions of apathy, including motivation, emotional response and goal-directed behaviour. In addition, NPI is also more effective than UPDRS-I in capturing the multifaceted nature of apathy. Thus, future research should consider integrating multiple apathy assessment tools and explore the onset and progression of neuropsychiatric symptoms in PD. Furthermore, the PPMI dataset does not include FLAIR images for all participants, which would be optimal for WMH assessments. While our T1w-based WMH measurements have been shown to hold strong correlations with FLAIR-based WMHs (r = 0.96, *P* < 0.001), future studies should also assess the relationship between apathy and FLAIR-based WMHs, which would be better indicators of the extent of WM damage.^44^

## Conclusion

Our study highlights the relationships between regional brain atrophy and WMH burden and future apathy in PD, providing important insights into the neural basis of apathy. Recognizing the contribution of WMHs to future apathy in PD provides an opportunity for timely interventions, improving the quality of life in patients.

## Supplementary Material

fcaf355_Supplementary_Data

## Data Availability

Data used in this article were obtained from the Parkinson's Progression Markers Initiative (PPMI) database (www.ppmi-info.org/data). For up-to-date information on the study, visit www.ppmi-info.org. PPMI is sponsored and partially funded by the Michael J Fox Foundation for Parkinson's Research and funding partners, including AbbVie, Avid Radiopharmaceuticals, Biogen, Bristol-Myers Squibb, Covance, GE Healthcare, Genentech, GlaxoSmithKline (GSK), Eli Lilly and Company, Lundbeck, Merck, Meso Scale Discovery (MSD), Pfizer, Piramal Imaging, Roche, Servier and UCB (www.ppmi-info.org/fundingpartners). The script for the image processing pipeline used to extract DBM and WMH measures is also open source and publicly available at https://github.com/VANDAlab/Preprocessing_Pipeline.
