# Supplementary material for: Apathy progression is associated with brain atrophy and white matter damage in Parkinson’s disease
Source: Brain Commun. 2025 Sep 13;7(5):fcaf355. doi: 10.1093/braincomms/fcaf355 (PMC12501503; doi:10.1093/braincomms/fcaf355)

# **Supplementary material**

Supplementary Table 1. Comparison of the demographics and clinical variables between the participants included and excluded in the study

| **Variable** | **Excluded** | **Included** | **P Value** |
| --- | --- | --- | --- |
| **Age** | 63.317 ± 10.093 | 62.42 ± 9.62 | 0.3 |
| **Sex(%female)** | 32 | 33.7 | 0.93 |
| **Education_Years** | 16.000 ± 3.379 | 15.99 ± 2.99 | 0.997 |
| **MOCA** | 26.556 ± 2.926 | 27.10 ± 2.26 | 0.296 |
| **GDS** | 1.789 ± 2.261 | 2.30 ± 2.48 | 0.045 |
| **STAI** | 60.90 ± 19.46 | 63.91 ± 18.12 | 0.209 |
| **Apathy Score** | 0.099 ± 0.300 | 0.24 ± 0.57 | 0.079 |
| **BMI** | 27.161 ± 4.4305 | 26.78 ± 4.78 | 0.493 |
| **HY Stage** | 1.618 ± 0.490 | 1.61 ± 0.50 | 0.913 |
| **UPDRS III** | 22.099 ± 8.469 | 21.41 ± 8.96 | 0.361 |

Supplementary Table 2. Hypothesis-based results for the longitudinal DBM analyses.

| **Hemisphere** | **Left** | | | | **Right** | | | |
| --- | --- | --- | --- | --- | --- | --- | --- | --- |
| Region Names | ID | T stats | P values | P_FDR_ | ID | T stats | P values | P_FDR_ |
| Middle Frontal Gyrus | 52 | 0.050 | 0.960 | 0.993 | 1 | -1.004 | 0.316 | 0.451 |
| **Nucleus Accumbens** | **55** | **-4.406** | **<0.001** | **<0.001** | **4** | **-3.305** | **0.001** | **0.005** |
| Orbitofrontal Cortex | 58 | -2.101 | 0.036 | 0.082 | 7 | -2.285 | 0.022 | 0.056 |
| Rostral Anterior Cingulate | 59 | -0.880 | 0.379 | 0.516 | 8 | 0.432 | 0.666 | 0.799 |
| **Superior Parietal Gyrus** | **60** | **-3.831** | **<0.001** | **0.001** | **9** | **-4.385** | **<0.001** | **<0.001** |
| Inferior Parietal Gyrus | 61 | -1.410 | 0.159 | 0.280 | 10 | 0.351 | 0.726 | 0.838 |
| **Putamen** | **72** | **-2.374** | **0.018** | **0.048** | **21** | **-3.435** | **0.001** | **0.004** |
| **Insula** | **74** | **-2.891** | **0.004** | **0.014** | **23** | **-2.540** | **0.011** | **0.037** |
| **Precuneus** | **82** | **-3.478** | **0.001** | **0.004** | 31 | -1.513 | 0.130 | 0.245 |
| **Precentral Gyrus** | 86 | -0.098 | 0.922 | 0.988 | **35** | **-3.032** | **0.002** | **0.010** |
| Caudal Midbrain | 93 | -0.610 | 0.542 | 0.678 | 42 | -0.625 | 0.532 | 0.678 |
| Inferior Frontal Gyrus | 95 | 1.964 | 0.050 | 0.106 | 44 | 1.349 | 0.177 | 0.296 |
| Superior Temporal Gyrus | 96 | -1.250 | 0.211 | 0.317 | 45 | -1.256 | 0.209 | 0.317 |
| **cerebellum gray matter** | 97 | -1.673 | 0.094 | 0.189 | **46** | **-2.494** | **0.013** | **0.038** |
| Caudate | 100 | 0.306 | 0.759 | 0.844 | 49 | 0.008 | 0.994 | 0.994 |

Supplementary Table 3. Exploratory results for the longitudinal DBM analyses.

| **Left Hemisphere** | | | | **Right Hemisphere** | | | |
| --- | --- | --- | --- | --- | --- | --- | --- |
| **ID** | **Region Names** | **T stats** | **PFDR** | **ID** | **Region Names** | **T stats** | **PFDR** |
|  |  |  |  | 3 | Inferior temporal | -2.5139 | 0.0382 |
| 55 | Accumbens Area | -4.4058 | <0.001 | 4 | Accumbens Area | -3.3049 | 0.0051 |
|  |  |  |  | 6 | Pericalcarine | -2.8933 | 0.0146 |
| 60 | Superior Parietal | -3.8314 | 0.0013 | 9 | Superior Parietal | -4.3848 | <0.001 |
| 63 | Lingual | -3.2757 | 0.0053 | 12 | Lingual | -4.113 | <0.001 |
|  |  |  |  | 15 | Medial Orbitofrontal | -2.6134 | 0.0306 |
| 69 | Para hippocampal | -3.0439 | 0.01 | 18 | Para hippocampal | -2.8694 | 0.015 |
| 72 | Putamen | -2.374 | 0.05 | 21 | Putamen | -3.4351 | 0.0041 |
| 74 | Insula | -2.8908 | 0.0146 | 23 | Insula | -2.5401 | 0.0366 |
| 76 | Basal Forebrain | -5.0842 | <0.000 | 25 | Basal Forebrain | -5.3788 | <0.001 |
| 77 | Ventral Diencephalon | -3.3631 | 0.0047 | 26 | Ventral Diencephalon | -3.6484 | 0.0025 |
| 79 | Middle Temporal | -4.9283 | <0.000 |  |  |  |  |
| 82 | Precuneus | -3.4779 | 0.0037 |  |  |  |  |
| 84 | Isthmus Cingulate | -3.359 | 0.0047 | 33 | Isthmus Cingulate | -3.1874 | 0.0067 |
|  |  |  |  | 35 | Precentral | -3.0322 | 0.01 |
| 88 | Fourth Ventricle | -3.895 | 0.0011 | 37 | Fourth Ventricle | -4.001 | <0.001 |
| 91 | Thalamus | -3.2696 | 0.0053 | 40 | Thalamus | -3.478 | 0.0037 |
| 92 | Lateral Ventricle | 3.3309 | 0.0049 | 41 | Lateral Ventricle | 3.0832 | 0.0091 |
|  |  |  |  | 43 | Cuneus | -2.788 | 0.0187 |
|  |  |  |  | 46 | Cerebellum Gray Matter | -2.4936 | 0.0392 |
| 99 | Hippocampus | -3.5901 | 0.0028 | 48 | Hippocampus | -4.3271 | <0.001 |

Supplementary Table 4. Exploratory results for the longitudinal WMH analyses.

| **Hemisphere** | **Left** | | **Right** | |
| --- | --- | --- | --- | --- |
| Region Names | T stats | P_FDR_ | T stats | P_FDR_ |
| Frontal Lobe | **2.560** | **0.047** | **2.950** | **0.029** |
| Temporal Lobe | 1.641 | 0.182 | 2.022 | 0.097 |
| Parietal Lobe | 0.172 | 0.864 | 0.282 | 0.864 |
| Occipital Lobe | -0.839 | 0.516 | -1.538 | 0.186 |

Supplementary Table 5. Exploratory results for the longitudinal DBM analyses with anxiety and depression scores, psychotropic medications, and vascular risk factors as covariates

| **Left Hemisphere** | | | | **Right Hemisphere** | | | |
| --- | --- | --- | --- | --- | --- | --- | --- |
| Region Names | ID | T stats | PFDR | Region Names | ID | T stats | PFDR |
| Accumbens Area | 55 | -3.3885 | 0.0007 |  |  |  |  |
| Superior Parietal | 60 | -3.0171 | 0.0026 | Superior Parietal | 9 | -3.4523 | 0.0006 |
| Lingual | 63 | -3.2182 | 0.0013 | Lingual | 12 | -4.0506 | 0.0001 |
| Para hippocampal | 69 | -2.9134 | 0.0036 | Para hippocampal | 18 | -2.7906 | 0.0053 |
| Basal Forebrain | 76 | -3.9734 | 0.0001 | Basal Forebrain | 25 | -3.8785 | 0.0001 |
| Ventral Diencephalon | 77 | -2.9231 | 0.0035 | Ventral Diencephalon | 26 | -3.3782 | 0.0007 |
| Middle Temporal | 79 | -4.3614 | <0.001 |  |  |  |  |
|  |  |  |  | Precentral | 35 | -2.6562 | 0.0079 |
| Fourth Ventricle | 88 | -3.5362 | 0.0004 | Fourth Ventricle | 37 | -3.4178 | 0.0006 |
| Lateral Ventricle | 92 | 3.1415 | 0.0017 | Lateral Ventricle | 41 | 2.7261 | 0.0064 |
|  |  |  |  | Cuneus | 43 | -2.6426 | 0.0083 |
|  |  |  |  | Hippocampus | 48 | -3.667 | 0.0002 |

Supplementary Figure 1. Association between Z-values from the ordinal analysis and corresponding t-statistics from the continuous analysis.(r = 0.92, p < 0.0001).


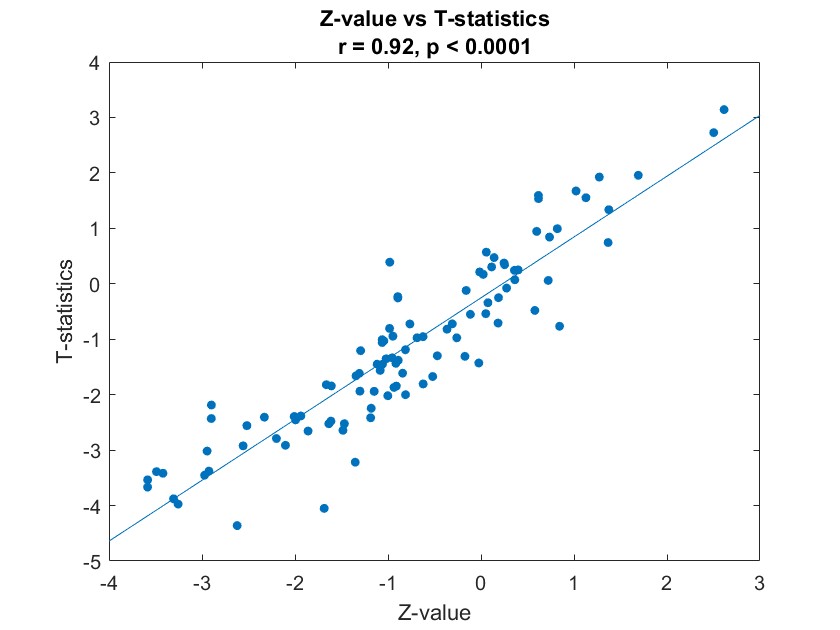

Supplement: fcaf355_Supplementary_Data [file fcaf355_supplementary_data.docx]
